# Functional Outcomes After Transoral Plus Lateral Pharyngotomy Approach for Advanced Oral and Oropharyngeal Tumors

**DOI:** 10.1002/oto2.35

**Published:** 2023-02-23

**Authors:** Sophia M. Colevas, Emily C. Merfeld, Zachary E. Pflum, Thomas G. Gessert, Aaron M. Wieland, Tiffany A. Glazer, Adam R. Burr, Paul M. Harari, Gregory K. Hartig

**Affiliations:** ^1^ Department of Surgery, Division of Otolaryngology–Head and Neck Surgery University of Wisconsin School of Medicine and Public Health Madison Wisconsin USA; ^2^ Department of Human Oncology University of Wisconsin School of Medicine and Public Health Madison Wisconsin USA

**Keywords:** head and neck cancer, lateral pharyngotomy, oropharyngeal cancer, radiation therapy, surgical salvage

## Abstract

**Objective:**

The aim of this study was to evaluate our institutional experience with the combined transoral plus lateral pharyngotomy (TO+LP) approach in a subset of patients with advanced or recurrent oral and oropharyngeal malignancy.

**Study Design:**

A retrospective study of procedures utilizing TO+LP for cancer resection between January 2007 and July 2019.

**Setting:**

Tertiary academic medical center.

**Methods:**

Thirty‐one patients underwent a TO+LP approach for the resection of oral and oropharyngeal tumors. Functional and oncologic outcomes were analyzed.

**Results:**

Eighteen (58.1%) patients were treated with TO+LP for recurrent disease. Twenty‐nine required free tissue transfer and 2 (6.5%) had positive margins. The median time to decannulation was 22 days (range 6‐100 days). Thirteen (41.9%) patients still required enteral feeding at their most recent follow‐up. Patients without a history of prior radiation were decannulated sooner (*p* = .009) and were less likely to require enteral feeding at the first postoperative follow‐up (*p* = .034) than those who had prior head and neck radiotherapy.

**Conclusion:**

A TO+LP approach can be used to achieve good functional and oncologic results for selected patients with advanced or recurrent oral and oropharyngeal cancer when minimally invasive options such as transoral robotic surgery, transoral laser microsurgery, or radiotherapy are not possible.

Transoral approaches are regularly employed for oral cavity malignancies and for early tumors of the oropharynx through the use of transoral robotic surgery (TORS) or transoral laser microsurgery (TLM) techniques. Larger or recurrent tumors involving both the oral cavity and oropharynx, as well as those of the oropharynx which are not amenable to TORS, TLM, or nonoperative options, are typically managed with the lip split and mandibulotomy approach. While this approach affords excellent visualization for the resection of large oropharyngeal tumors, it comes with an increased risk of morbidity, with reported mandibulotomy‐related complication rates of up to 11%.^1^, ^2^

The transcervical approach to the oropharynx, known as the lateral pharyngotomy,^3^, ^4^ has been well described for the treatment of early‐stage tumors.^5^, ^6^, ^7^ With the advent of TORS, TLM, and improvements in primary radiotherapy therapy, it has been utilized less frequently in recent years.^8^ However, in select patients, a combined transoral and lateral pharyngotomy (TO+LP) approach remain useful. In our institution, we employ this approach for many larger or recurrent tumors involving both the oral cavity and pharynx that would otherwise be managed with a mandibulotomy with lip split when a TORS, TLM, or nonoperative approach is not possible.

Most existing reports of functional and oncologic outcomes after lateral pharyngotomy are dominated by patients with smaller and previously untreated tumors.^9^, ^10^, ^11^, ^12^ Furthermore, most utilized primary closure^5^, ^6^, ^7^, ^8^, ^9^ or local flaps^11^ to reapproximate the pharynx, with very few reported free flaps for reconstruction.^6^ Due to the limited published data assessing outcomes among patients who undergo lateral pharyngotomy and free flap reconstruction for advanced or recurrent oropharyngeal malignancies, the aim of this study was to evaluate our institutional experience with the combined TO+LP approach in this subset of patients.

## Materials and Methods

We obtained Institutional Review Board approval and queried the University of Wisconsin Head and Neck Cancer Database for all patients treated for head and neck cancer who underwent regional or free tissue transfer and lateral pharyngotomy between January 1, 2007 and July 1, 2019. Patients who underwent laryngectomy, maxillectomy, parotidectomy, or skull base resections were excluded. From the remaining list, patients with oral cavity or oropharyngeal malignancy who were treated with a combined TO+LP approach were included.

**Figure 1:**
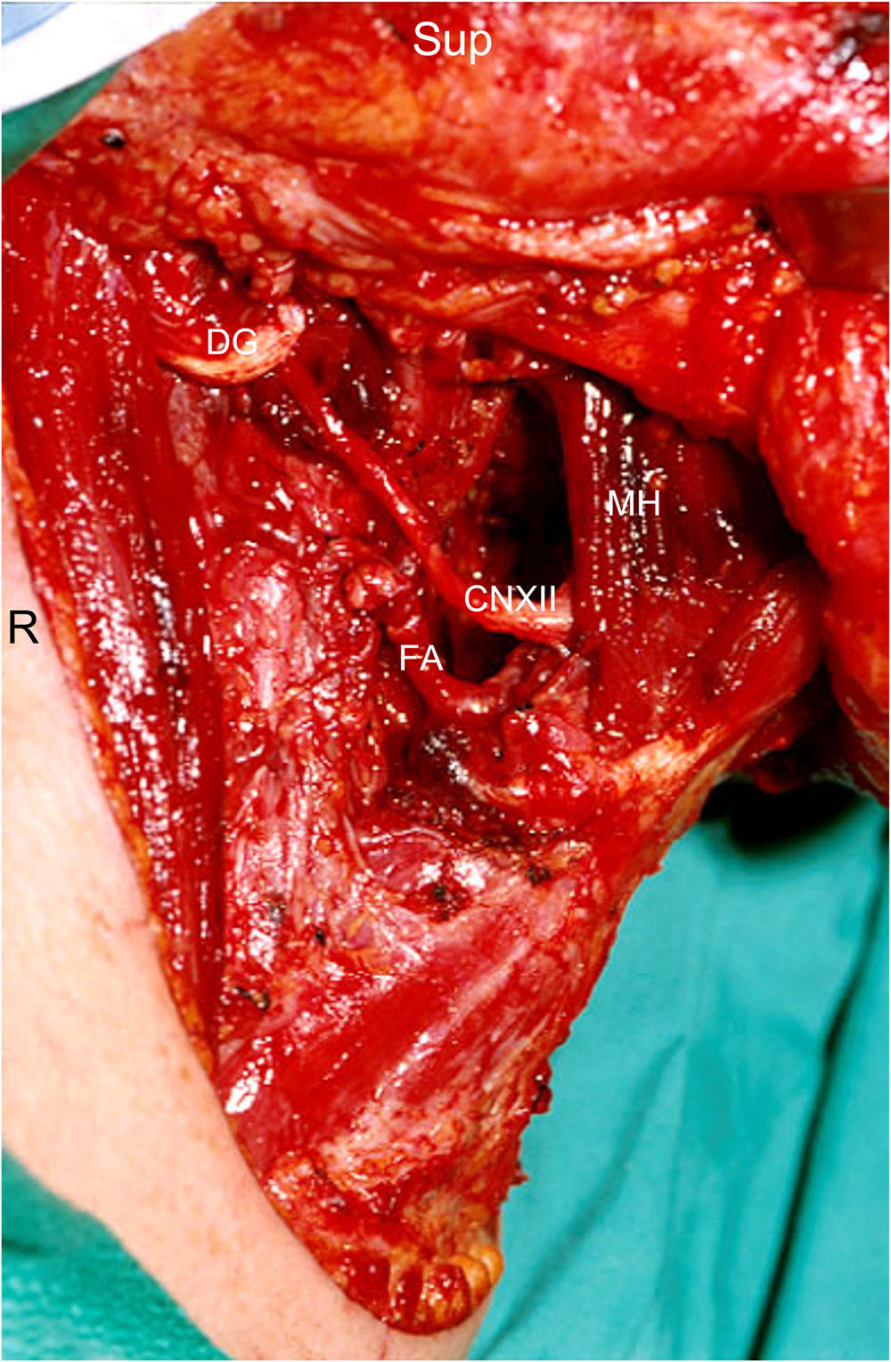
Right lateral pharyngotomy defect after tumor resection. CN XII, cranial (hypoglossal) nerve; DG, cut edge of the posterior belly of digastric muscle; FA, facial artery; MH, mylohyoid muscle; R, right; Sup, superior.

**Figure 2:**
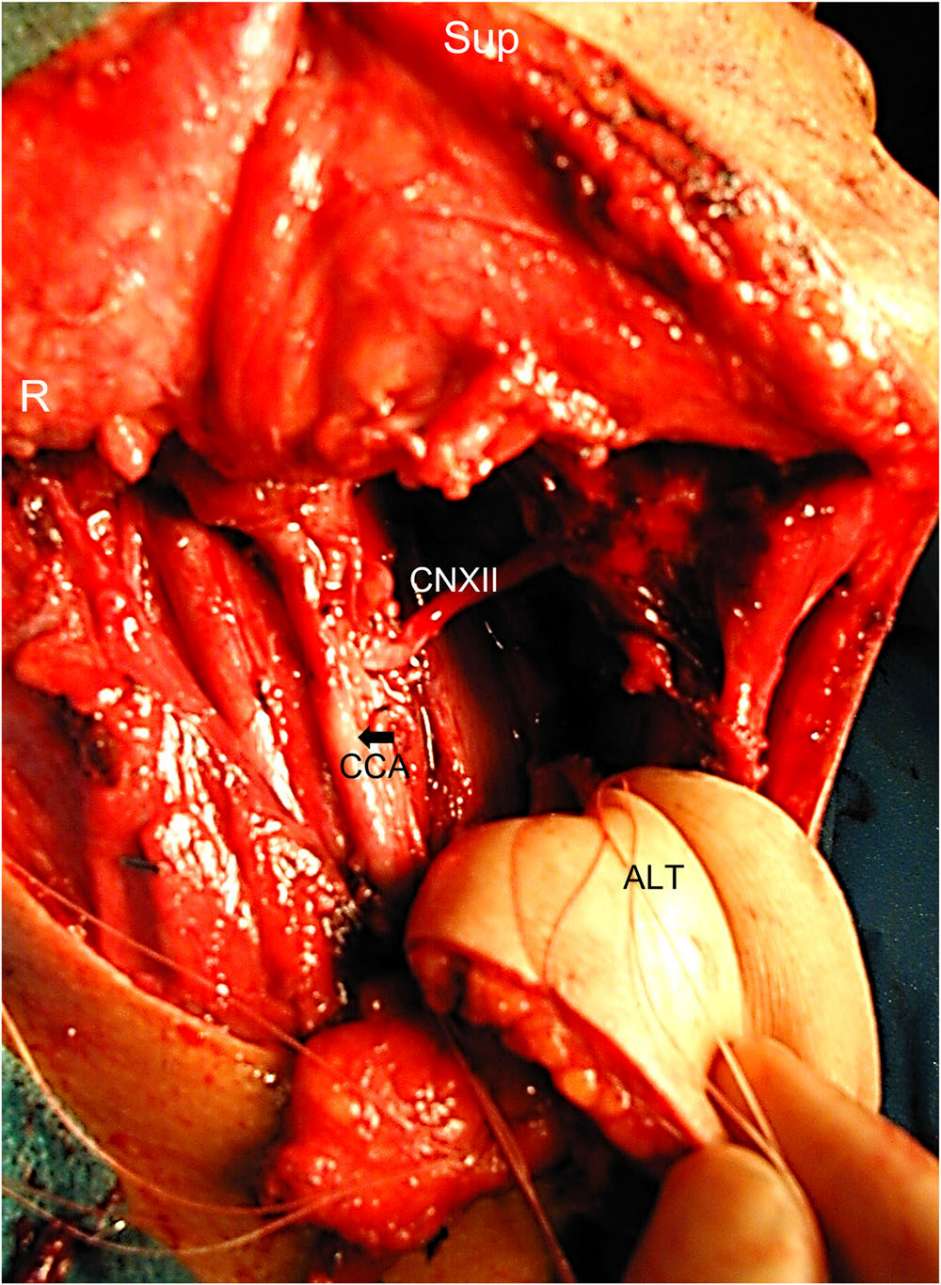
Transcervical anterolateral thigh flap inset into right lateral pharyngotomy defect. ALT, inferior inset of anterolateral thigh flap; CCA with arrow, common carotid artery; CN XII, cranial (hypoglossal) nerve; R, right; Sup, superior.

The technique for the TO+LP begins with the removal of the submandibular gland and level one lymph nodes with ligation of the facial artery and common facial vein branches. Next, cranial nerve 12 and the superior laryngeal nerve are freed from surrounding tissues, the lingual artery is ligated, the digastric muscle is divided, and the greater horn of the hyoid on the side of the resection is removed. With this, the pharynx is separated from the vasculature and the cranial nerve branches. With a malleable ribbon retractor placed lateral to the pharyngeal constrictors, the anterior and superior aspects of the resection can be accomplished transorally and mobilized through the pharyngotomy. The remaining resection is then accomplished transcervically. This often requires mobilization of the resection around the hypoglossal nerve to preserve this structure (Figure 1). Reconstruction, typically with free tissue transfer, proceeds in the reverse order with the inferior and posterior aspects of the flap inset accomplished through the lateral pharyngotomy and the superior and anterior elements accomplished transorally (Figure 2). Full inset is accomplished prior to revascularization of free tissue transfers.

Patient records were reviewed for demographic data, staging, treatment, postoperative course, recurrence, and survival information. The initial tumor stage was determined using the Tumor, Node, and Metastasis/American Joint Committee on Cancer 8th edition. Complications directly relating to surgery were recorded if they occurred within 1 year after surgery. Postoperative swallow function was recorded based on first (1‐2 week) postoperative follow‐up notes from the surgeon or speech‐language pathologist, as available. The most recent swallow function was recorded based on the last documented visit with an oncologist or speech‐language pathologist in which the swallow function was discussed. The duration of tracheostomy dependence was calculated as the time elapsed from tracheostomy placement to decannulation. The duration of enteral feeding was calculated as the time elapsed between surgery and removal of all mechanisms of enteral feeding (nasogastric tube or gastrostomy tube [g‐tube]).

Independent, 2‐sample *t* tests and *χ* ^2^ tests of independence were used to compare tumor size, margin status, functional outcomes, complication rates, and time to most recent swallow assessment between patients who had undergone radiation therapy at some time prior to TO+LP, versus those with no prior radiotherapy. If data did not meet the statistical assumptions for parametric testing, a Wilcoxon‐Mann‐Whitney rank sum test was performed. Kaplan‐Meier survival curves were generated to analyze disease‐free survival for patients who had surgical resection via TO+LP approach for their initial disease presentation and for those treated for a recurrence. Statistical analysis was performed using SPSS, version 26 (SPSS, Inc and IBM Company) and a *p* value of less than .05 was considered statistically significant.

## Results

### Demographics

Between 2007 and 2019, 31 patients underwent surgical resection of an oral or oropharyngeal tumor using a TO+LP approach. Patient ages ranged from 42 to 73 years (mean 60.1 years). The mean Charlson comorbidity index was 4.4, and 24 (77.4%) patients were current or former smokers with an average of 29.3 pack years. Other demographic details are shown in Table 1.

**Table 1:** Demographics and Disease Risk Factors for Patients Undergoing Resection of Oral and Pharyngeal Malignancies Via Lateral Pharyngotomy

Demographics	n (%)
Gender	
Male	24 (77.4)
Female	7 (22.6)
Age	
40‐49	3 (9.7)
50‐59	10 (32.3)
60‐69	16 (51.6)
70+	2 (6.5)
Charleson comorbidity index	
0‐2	1 (3.2)
3‐4	21 (67.7)
5‐6	5 (16.1)
7‐8	2 (6.5)
9+	2 (6.5)
Smoking history	
None	7 (22.6)
Former	9 (29.0)
Current	15 (48.4)
Alcohol use	
None	6 (18.8)
Former, heavy	5 (15.6)
Current	17 (53.1)

### Disease Characteristics

An overview of patient disease characteristics and history is shown in Table 2. The tongue base was the most involved disease site (21/31, 67.7%). Twenty patients (64.5%) had stage IV disease at the initial diagnosis. Eighteen (58.1%) patients had surgery with a TO+LP approach for recurrent disease, with a mean time of 2.26 years between initial treatment and the TO+LP for recurrence. Seventeen (54.8%) patients received head and neck radiotherapy prior to undergoing the TO+LP procedure, either as part of the initial treatment for their cancer, which then recurred or for a separate primary tumor of the head and neck. The most common histopathologic tumor type was squamous cell carcinoma (25/31, 80.6%) (Table 2).

**Table 2:** Characteristics of Patient Disease Including Tumor Site, Stage, and Prior Treatment

Disease characteristics	n (%)
Tumor sites	
Tongue base	13 (41.9)
Oral tongue, tongue base	4 (12.9)
Tonsil, tongue base	4 (12.9)
Oral tongue	3 (9.7)
Tonsil	2 (6.5)
Floor of mouth	1 (3.2)
Oral tongue, floor of mouth	1 (3.2)
Oral tongue, retromolar trigone	1 (3.2)
Parapharyngeal space, pharynx	1 (3.2)
Soft palate	1 (3.2)
The stage at initial diagnosis	
T stage	
T1	2 (6.5)
T2	14 (45.2)
T3	3 (9.7)
T4	1 (3.2)
T4a	10 (32.3)
Unable to determine	1 (3.2)
Overall stage	
I	1 (3.2)
II	8 (25.8)
III	1 (3.2)
IVA	19 (61.3)
IVB	1 (3.2)
Unable to determine	1 (3.2)
Histology	
Squamous cell carcinoma	25 (80.6)
Adenocarcinoma	2 (6.5)
Adenoid cystic carcinoma	1 (3.2)
Sarcomatoid carcinoma	1 (3.2)
Mucoepidermoid carcinoma	1 (3.2)
Basaloid squamous cell carcinoma	1 (3.2)
p16 status	
Positive	6 (19.4)
Negative	22 (71.0)
Unknown	3 (9.7)
Treatment history	
Recurrent disease at presentation	18 (58.1)
Prior RT	16 (51.6)
RT alone	10 (32.3)
Surgery + RT	7 (22.6)
Prior surgery alone	2 (6.5)
Initial presentation	13 (41.9)

Abbreviation: RT, radiation therapy.

### Treatment and Operative Course

The mean maximum dimension of the tumors resected via TO+LP was 3.4 cm. Two (6.5%) patients had positive margins noted on the final pathology (Table 3). Twenty‐nine (93.5%) patients required free flap reconstruction of the TO+LP defect. Radial forearm and anterolateral thigh free flaps were the most used reconstructive methods at 58.1% (18/31) and 38.7% (12/31), respectively, with 1 patient requiring both types due to the size of the defect. One patient (3.1%) required regional tissue transfer for reconstruction. One patient underwent primary closure without the need for additional reconstruction. There were no instances of flap failure. Twelve patients, 1 of whom had received prior radiotherapy, went on to have adjuvant radiotherapy after surgical resection via TO+LP. Of those who received surgical resection followed by adjuvant radiotherapy, 7 had oral cavity primary tumors. Of the remaining 5 patients, 3 received primary surgery followed by adjuvant radiotherapy for minor salivary gland tumors of the tongue base, 1 underwent primary TORS resection that was converted to open via the TO+LP approach, and 1 patient had been previously irradiated.

**Table 3:** Summary of Pathology Results Including Resected Tumor Size, Margin Status, and Distance From Closest Margin for All Patients, Patients With a History of Prior RT, and Patients Without a History of Prior RT

	All patients	No prior RT	Prior RT	*p* value
Pathology				
Number of patients	31	14	17	
Mean tumor size (cm)	3.43 ± 1.49	3.88 ± 1.82	3.06 ± 1.08	**.133**
Margins				.185
Positive	2 (6.5)^a^	0 (0)	2 (11.8)	
Negative (mm)	29 (93.6)	14 (100)	15 (88.2)	
0.5‐1	11 (37.9)	7 (50.0)	4 (26.7)	
1.01‐2	6 (20.7)	1 (7.1)	5 (33.3)	
2.01‐3	3 (10.3)	1 (7.1)	2 (13.3)	
3.01‐4	2 (6.9)	1 (7.1)	1 (6.7)	
4.01‐5	3 (10.3)	2 (14.3)	1 (6.7)	
>5	1 (3.4)	0 (0)	1 (6.7)	
Distance to margin				
Unknown	3 (10.3)	2 (14.3)	1 (6.7)	

Unpaired 2‐sample *t* tests and *χ* ^2^ tests of independence were used to compare the differences between those with and without a history of RT prior to surgery.

The bold value indicated statistical significance (*p* < .05).

Abbreviation: RT, radiation therapy.

^a^ Data are reported as “Count (percentage)” for categorical variables.

### Postoperative Course

Data relating to the postoperative courses are shown in Table 4. Median postoperative admission length was 9 days (range 2‐18), with 5 patients requiring readmission. Readmission reasons included 2 instances of neck surgical site infection without fistula requiring IV antibiotics, and 1 instance each of recurrent disease, chest pain, and aspiration pneumonia. The overall 1‐year postoperative complication rate was 32%, with 10 patients having at least 1 complication. The most common postoperative complication was aspiration pneumonia. Only 1 (3.2%) patient developed a salivary fistula.

**Table 4:** Summary of Postoperative Course and Functional Outcomes for All Patients, Patients With a History of Prior RT, and Patients Without a History of Prior RT

	All patients	No prior RT	Prior RT	*p* value
*Postoperative course*				
Number of patients	31	14	17	
Median postoperative admission duration (d)	9 (2‐18)^a^	9 (7‐18)	8 (2‐11)	.163
Patients requiring readmission	5 (16.1)^b^	2 (14.3)	3 (17.6)	.800
*Postoperative complications*				
Aspiration pneumonia	6 (19.4)	3 (21.4)	3 (17.6)	.791
Infection	4 (12.9)	2 (14.3)	2 (11.8)	.835
Salivary fistula	1 (3.2)	1 (7.1)	0 (0.0)	.263
Secondary trach/intubation	1 (3.2)	1 (7.1)	0 (0.0)	.263
Seroma	1 (3.2)	1 (7.1)	0 (0.0)	.263
Chyle leak	1 (3.2)	0 (0.0)	1 (5.9)	.356
Nerve injury	0 (0.0)	0 (0.0)	0 (0.0)	
Bleeding/hematoma	0 (0.0)	0 (0.0)	0 (0.0)	
*Tracheotomy*				
Patients with tracheotomy	30 (96.8)	13 (92.9)	17 (100)	
Median time to decannulation (d)	22 (6‐100)	13 (6‐27)	32 (9‐100)	**.009**
Patients never decannulated	1 (3.2)	0 (0.0)	1 (5.9)	
Unable to determine trach status	2 (6.5)	1 (7.1)	1 (5.9)	
*Enteral feeding*				
Median time to removal of enteral feeds (NGT or g‐tube) (d)	71 (11‐451)	82 (11‐451)	68.5 (15‐136)	.209
*Swallow function—discharge*				
Enteral feed (NGT or g‐tube)	30 (96.8)	13 (92.9)	17 (100)	
Unable to determine	1 (3.2)	0 (0.0)	1 (5.9)	
*Swallow function—first follow‐up*				
Enteral feed (NGT or g‐tube)	17 (54.8)	5 (35.7)	12 (70.6)	**.034**
Soft	12 (38.7)	9 (64.3)	3 (17.6)	
Liquids	1 (3.2)	0 (0.0)	1 (5.9)	
Unable to determine	1 (3.2)	0 (0.0)	1 (5.9)	
Regular	0 (0.0)	0 (0.0)	0	
*Swallow function—last follow‐up*				
Median time to last swallow follow‐up (mo)	18 (3.0‐87.8)	28.2 (3.3‐8.8)	12.6 (3.0‐82.2)	.313
Enteral feed (NGT or g‐tube)	13 (41.9)	3 (21.4)	10 (58.8)	.073
Regular	7 (22.6)	5 (35.7)	2 (11.8)	
Soft	5 (16.1)	3 (21.4)	2 (11.8)	
Liquids	4 (12.9)	3 (21.4)	1 (5.9)	
Required laryngectomy	1 (3.2)	0 (0.0)	1 (5.9)	
Unable to determine	1 (3.2)	0 (0.0)	1 (5.9)	

Unpaired 2‐sample *t* tests and *χ* ^2^ tests of independence were used to compare the difference in means for various function outcomes between those with and without a history of RT prior to surgery.

Bold values indicated statistical significance (*p* < 0.05).

Abbreviations: g‐tube, gastrostomy tube; NGT, nasogastric tube; RT, radiation therapy.

^a^ Data are reported as “Median (range)” for continuous variables.

^b^ Data are reported as “Count (percentage)” for categorical variables.

### Functional Outcomes

Thirty (96.8%) patients had a tracheostomy placed during surgery and 1 patient did not require a tracheostomy. The median time to decannulation was 22 days (range 6‐100). One patient was never decannulated (Table 4).

Twenty‐five (80.7%) patients were discharged with a g‐tube. Five (16.1%) patients did not receive an immediate postoperative g‐tube but were discharged with enteral feeding via nasogastric tube. Thirteen (41.9%) patients never had their g‐tube removed. For those patients who temporarily required enteral feeding, the median time to discontinuation of enteral feeding was 71 days (range 11‐451). A summary of patient swallow function (type of feeding tolerated) at the first (1‐2 week) postoperative follow‐up visit and at the most recent follow‐up visit is shown in Table 4. The mean time to the most recent assessment of swallow function was 27.0 months (median 18.0 months, range 3.0‐87.8).

### Recurrence and Survival

**Figure 3:**
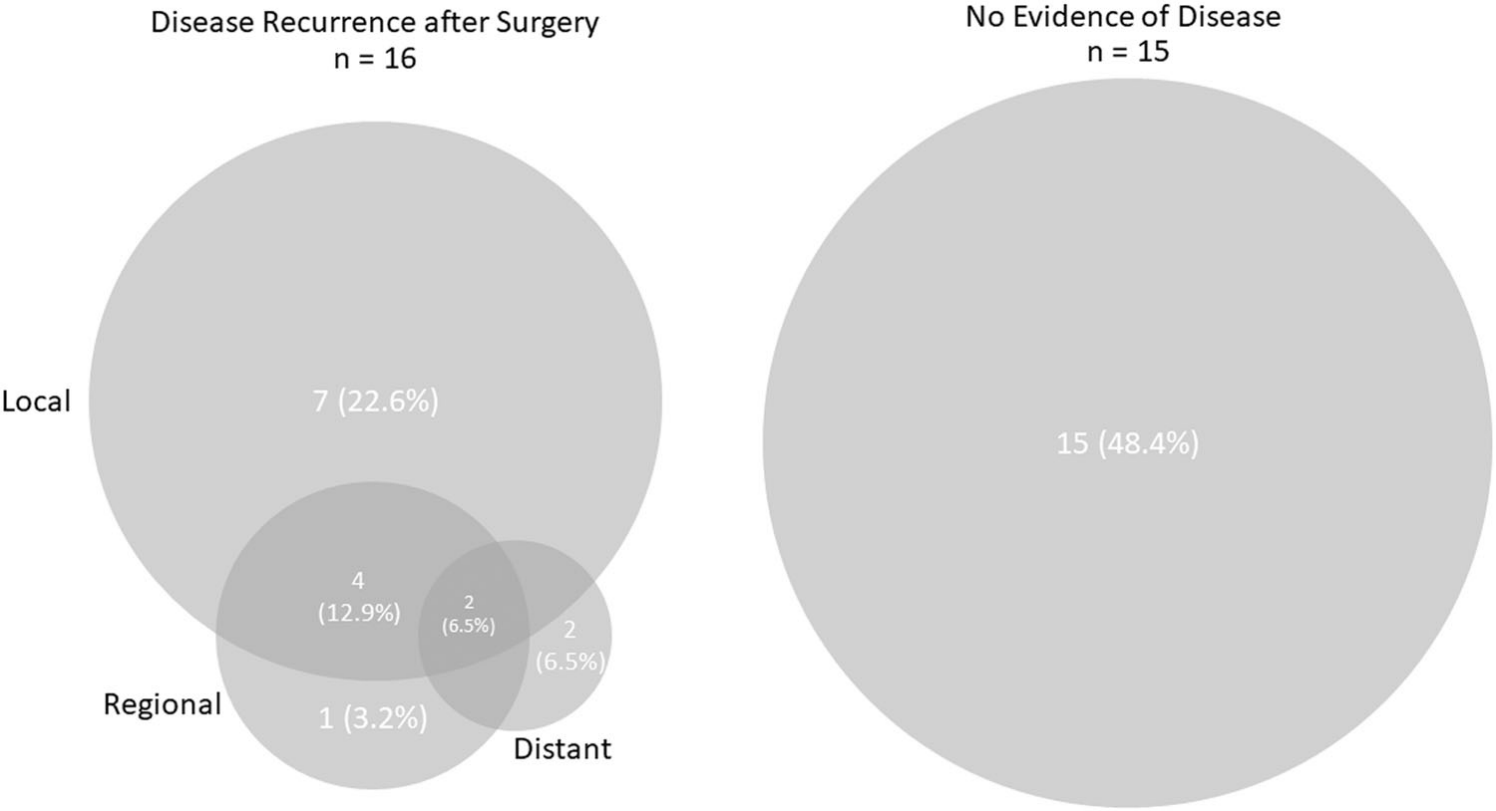
Patterns of recurrence after lateral pharyngotomy for all patients.

Fifteen (48.4%) patients had no recurrence after resection via TO+LP; 2 additional patients experienced isolated distant metastases, for a total locoregional control rate of 54.8% (Figure 3). The median duration of disease‐free follow‐up was 28.2 months (range 3.3‐70.7). The median time to recurrence was 4.8 months (range 0.5‐75.8).

**Figure 4:**
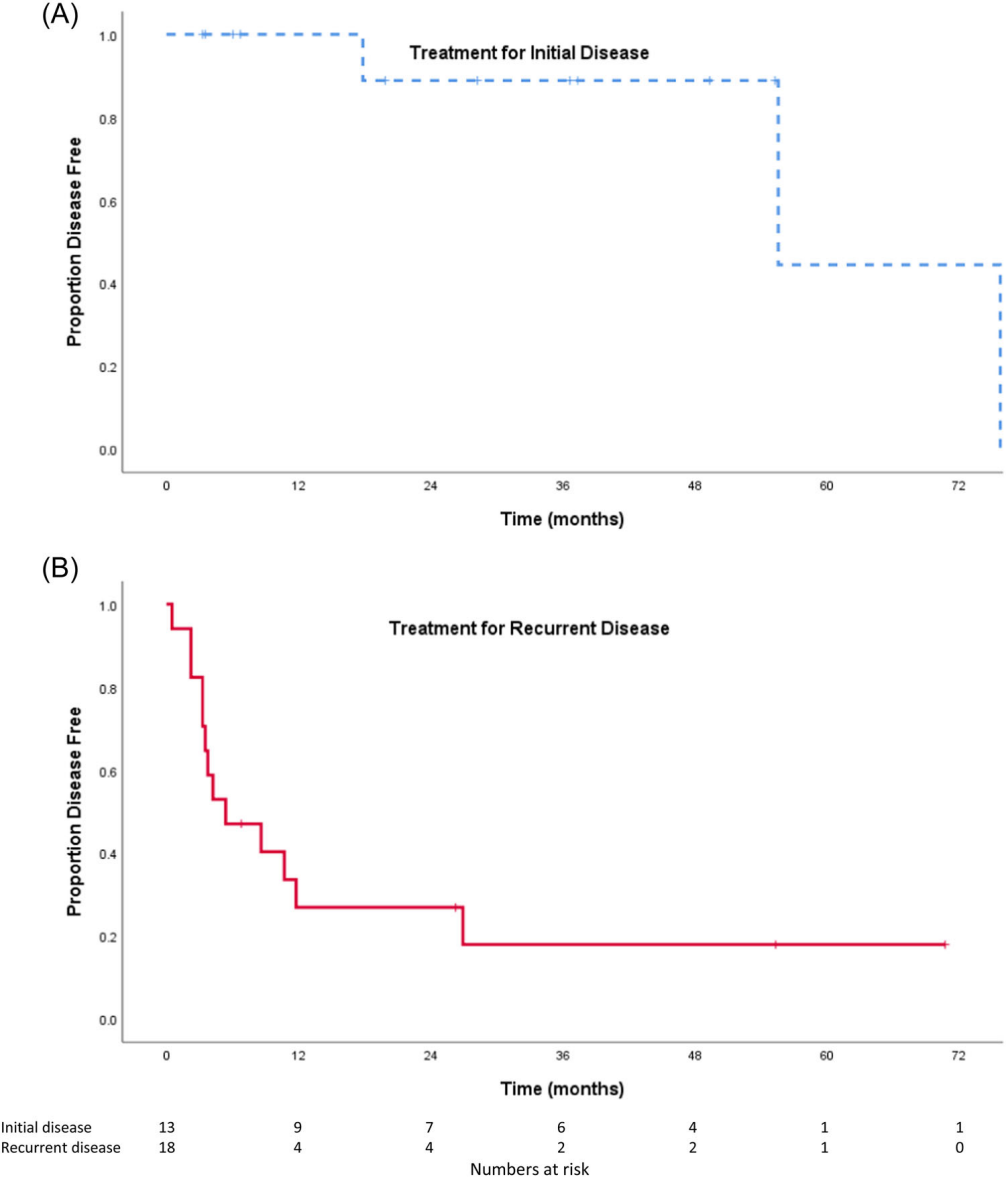
Kaplan‐Meier analysis of disease‐free survival for patients treated with lateral pharyngotomy for (A) initial disease and (B) recurrent disease.

Disease‐free survival was analyzed for subjects who had surgical resection via TO+LP for their initial disease presentation and for those treated for recurrence (Figure 4). At the median follow‐up time of 28.2 months, disease‐free survival was 89% among patients treated for initial disease and 18% among patients treated for a recurrence.

### History of Prior Radiation Therapy

Patients with prior radiotherapy were, on average, decannulated 22 days later than those without a history of radiation to the head and neck (*p* = .009). There was a significant association between a history of prior radiation and swallow function at first postoperative follow‐up, with 75% (12/16) of patients with prior radiotherapy requiring enteral feeding at first follow‐up, compared with 36% (5/14) of patients without prior radiotherapy (*Χ* ^2^_2_ = 6.8, *p* = .034).

While there was a wide range in time to the most recent assessment of swallow function across all patients, there was no significant difference in follow‐up time detected between those with a history of radiation therapy and those without a history of radiation therapy (*p* = .313). The association between history of radiation and swallow function at the last documented follow‐up approached significance when all categories of feeding type tolerated were included (*Χ* ^2^_3_ = 7.0, *p* = .073). While there was not a significant difference in the time to removal of enteral feeding between groups, those with prior radiation had a significantly lower chance of ever having enteral feeding removed, with 69% (11/16) of patients with prior radiotherapy still requiring enteral feeding at last documented follow‐up, compared with 21% (3/14) of patients without a history of radiation (*Χ* ^2^_1_ = 5.4, *p* = .020). There were no significant differences in any other functional outcomes or the rate of postoperative complications between groups.

## Discussion

Most patients treated with TO+LP had larger tumors, a history of radiation, or recurrent disease. Using the TO+LP approach for resection, an excellent negative margin rate was achieved in this population. Furthermore, postoperative complications were limited, and functional outcomes were comparable to those reported in prior studies of early‐stage patients.

### Margin Status After TO+LP

In the current study, negative margins were achieved using the TO+LP approach in most patients, many of whom had been previously irradiated. Surgical planes following high‐dose radiotherapy can be obscured by soft tissue fibrosis and telangiectasias. Therefore, complete resection of the recurrent tumor frequently requires radical excision with wide clinical margins.^13^ Our negative margin rate of 94% (88% among previously irradiated patients) compares favorably with rates of 61% to 97% reported in the literature.^5^, ^6^, ^7^, ^9^, ^11^, ^12^, ^14^, ^15^ This speaks to the quality of exposure achievable with the TO+LP even in more technically challenging situations.

### Oncologic Outcomes After TO+LP

While the rate of disease‐free survival at 28 months after LP for our entire cohort was 47%, it was 89% among patients treated for an initial diagnosis of head and neck cancer. This compares favorably with disease‐free survival rates of 61% to 86% following lateral pharyngotomy among other previously untreated cohorts.^7^, ^9^, ^11^ Disease‐free survival rates for patients treated for recurrence were 29.4%, which is comparable to rates of 23% to 43% reported in the current literature for surgical salvage of recurrent oral cavity or oropharyngeal tumors.^16^, ^17^, ^18^, ^19^, ^20^, ^21^

### Postoperative Complications After TO+LP

Postoperative complication rates in the current study compared favorably with other studies, despite the heavily pretreated patient population. In a study involving the use of the lateral pharyngotomy approach in a cohort of previously untreated T1 and T2 tumors of the pyriform sinus, Holsinger et al report 1 (3%) postoperative complication of death related to salivary fistula.^5^ Most other studies report postoperative complication rates after lateral pharyngotomy between 26% and 39%^6^, ^9^, ^22^ which is consistent with our rate of 32%. It is notable that our rate of complications remains consistent with these other studies despite the increased number of advanced tumors and prior radiotherapy in our patient cohort. Within our cohort, the rate of operative complications, such as infection, fistula, or chyle leak, was 25.8%, with only a 19.4% rate of complications specifically related to pharyngeal resection (aspiration pneumonia). Patients treated at our institution were discharged after a median of 9 days (range 2‐18), compared to a range of 12 to 19 days reported in other studies.^5^, ^9^, ^12^, ^22^

Treatment of recurrent head and neck cancer is challenging and can be associated with substantial morbidity. Postoperative complication rates of 17% to 67% in this setting have been reported,^20^, ^21^, ^23^, ^24^, ^25^, ^26^ with postoperative admissions up to 12 days.^19^ Comparatively, the complication rate in our cohort of patients with recurrent head and neck cancer and prior radiotherapy undergoing TO+LP was 35%, and the median postoperative admission length was only 8 days.

### Functional Outcomes After Lateral Pharyngotomy

Duration of postoperative tracheostomy and enteral feeding are important indicators of functional outcome after pharyngeal tumor resection. Prior studies of the lateral pharyngotomy for resection of untreated, mostly T1 to T2 tumors report almost exclusively temporary tracheostomy and enteral feeding requirements. Median time to decannulation between 3 and 8 days^5^, ^9^, ^12^, ^22^ have been reported for patients whose tumor defect was able to be closed primarily. Lim et al describe a cohort of mostly T1 to T2 hypopharyngeal tumors that were treated with primary resection via lateral pharyngotomy where 70% of patients required reconstruction with a radial forearm free flap.^6^ Median duration of tracheostomy dependence in this group of patients was 28 days (range 11‐113), with 1 patient permanently tracheostomy dependent. This compares to our patient cohort, in which 29 out of 31 patients required free flap reconstruction, where the median time to decannulation was 22 days (range 6‐100).

Other studies report average postoperative enteral feeding durations of 9 to 12 days,^5^, ^9^, ^22^ with very few patients requiring g‐tube. Bertolin et al report that 1 patient out of 64 treated primarily for early to intermediate‐stage oropharyngeal carcinoma required g‐tube placement due to significant swallowing dysfunction. In a Laccourreye et al paper, where only 7% of subjects had prior radiotherapy, 6.5% of patients required g‐tube placement for up to 12 months.^22^ While 96.8% of patients in the current study required enteral feeding at discharge, and 54.8% of patients still required enteral feeding at their first postoperative visit, it is important to highlight that these rates are representative of a heavily pretreated population. Only 5 out of 14 patients without prior radiotherapy required enteral feeding at the first postoperative visit. Furthermore, 12 of these 14 patients received adjuvant radiotherapy following TO+LP, which likely contributed to the decision to maintain enteral feeding.

Patients with a history of prior radiotherapy had longer median times to decannulation and a higher likelihood of requiring enteral feeding at the first postoperative visit and at the last follow‐up. In our study, 41% of patients with prior radiotherapy were nondependent on enteral feeding at the time of the last follow‐up. This compares favorably to other studies, which have reported long‐term g‐tube dependence rates of 30% to 65% after salvage surgery for oral cavity and oropharyngeal tumors.^19^, ^24^, ^27^, ^28^

While the TO+LP approach is applicable for many patients who have combined oral/oropharyngeal tumors or combined oropharyngeal/hypopharyngeal tumors, there are situations where a lip split with mandibulotomy or lingual release is preferred. For tumors involving the masticator space or for those requiring mandibular resection, we find the view afforded with the TO+LP approach is limiting and can compromise confident resection. Also, for tumors requiring a subtotal or total glossectomy, a full lingual release is employed. Ideal tumors for the TO+LP approach are those that involve the middle and posterior thirds of the tongue, or the lateral oropharynx without extension to the retromolar area and masticator space. In today's era, the TO+LP approach will be most applicable for patients who have failed TORS, TLM, or nonoperative management of human papillomavirus‐associated oropharyngeal tumors and who require salvage resection with smaller volume‐free tissue transfer.

### Limitations

This study has several limitations. First, our cohort of patients was heterogeneous, with advanced or recurrent tumors requiring individualized approaches for management. We were, therefore, unable to include a direct matched comparison to other surgical approaches used at our institution, such as the mandibulotomy with lip split. Second, this was a retrospective study where outcomes are reported in a primarily descriptive manner, and we were limited by the information available in the medical record. In some cases, relevant information, such as radiation dose, complete histopathologic data, and speech and swallow follow‐up, could not be obtained in full for each patient. Additionally, markers such as duration of tracheostomy and enteral feeding are incomplete ways of assessing function. Quantitative measures, such as the PSSHN and MDADI exist, but could not be used due to the retrospective nature of this study.

## Conclusion

This study demonstrates that the combined TO+LP approach to oral and oropharyngeal tumor resection can be used for select patients with advanced or recurrent cancer with good functional and oncologic results. This includes its application to patients with recurrent disease as well as those with a history of prior radiation where the prognosis is generally poor. In the setting of surgical salvage, the TO+LP approach can be used to obtain similar margin status and postoperative complication rates as reported in studies of early disease. While our locoregional control after TO+LP for salvage was inferior to many studies describing outcomes after lateral pharyngotomy for early‐stage disease, disease‐free survival was comparable to rates reported in studies of salvage surgery for recurrent head and neck cancer. These results support the use of a combined TO+LP approach to resection and reconstruction in this subset of patients with recurrent and advanced oral and oropharyngeal malignancy.

## Author Contributions

**Sophia M. Colevas**, study design, data collection, analysis, manuscript drafting and revision, final approval; **Emily C. Merfeld**, study design, data collection, analysis, manuscript drafting and revision; **Zachary E. Pflum**, study design, data collection, analysis, manuscript drafting and revision; **Thomas G. Gessert**, critical revision of the manuscript; **Aaron M. Wieland**, critical revision of the manuscript; **Tiffany A. Glazer**, critical revision of the manuscript; **Adam R. Burr**, critical revision of the manuscript; **Paul M. Harari**, critical revision of the manuscript, final approval of the version to be published; **Gregory K. Hartig**, study conception and design, critical revision of the manuscript, final approval of the version to be published.

## Disclosures

### Competing interests

The authors have no conflicts of interest to disclose.

## Funding source

This work was supported in part by the NIH P50 DE026787—University of Wisconsin Head and Neck SPORE Grant.

## References

[1] Dziegielewski PT , Mlynarek AM , Dimitry J , Harris JR , Seikaly H . The mandibulotomy: friend or foe? Safety outcomes and literature review: mandibulotomy safety outcomes. Laryngoscope. 2009;119(12):2369‐2375. 10.1002/lary.20694 19806651

[2] Dziegielewski PT , O'Connell DA , Rieger J , Harris JR , Seikaly H . The lip‐splitting mandibulotomy: aesthetic and functional outcomes. Oral Oncol. 2010;46(8):612‐617. 10.1016/j.oraloncology.2010.05.006 20619720

[3] Pilcher LSI . On lateral pharyngotomy for the extirpation of malignant tumors of the tonsillar region. Ann Surg. 1886;4(2):139‐144. 10.1017/s0022215121000876 PMC1430892 17856086

[4] Trotter W . A method of lateral pharyngotomy for the exposure of large growths of the epilaryngeal region. Proc R Soc Med. 1920;13:196‐198. 19981095 10.1177/0035915720013007105 PMC2152878

[5] Holsinger FC , Motamed M , Garcia D , Brasnu D , Ménard M , Laccourreye O . Resection of selected invasive squamous cell carcinoma of the pyriform sinus by means of the lateral pharyngotomy approach: the partial lateral pharyngectomy. Head Neck. 2006;28(8):705‐711. 10.1002/hed.20375 16786602

[6] Lim YC , Jeong HM , Shin HA , Choi EC . Larynx‐preserving partial pharyngectomy via lateral pharyngotomy for the treatment of small (T_1∼2_) hypopharyngeal squamous cell carcinoma. Clin Exp Otorhinolaryngol. 2011;4(1):44‐48. 10.3342/ceo.2011.4.1.44 21461063 PMC3062227

[7] Remmert C , Mansour N , Hofauer B , et al. Pharyngotomy in head and neck squamous cell carcinoma: functional and oncological aspects. Acta Otolaryngol. 2017;137(12):1281‐1287. 10.1080/00016489.2017.1355564 28743201

[8] Cracchiolo JR , Baxi SS , Morris LG , et al. Increase in primary surgical treatment of T1 and T2 oropharyngeal squamous cell carcinoma and rates of adverse pathologic features: National Cancer Data Base. Cancer. 2016;122(10):1523‐1532. 10.1002/cncr.29938 26970050 PMC4860079

[9] Bertolin A , Ghirardo G , Lionello M , Giacomelli L , Lucioni M , Rizzotto G . Lateral pharyngotomy approach in the treatment of oropharyngeal carcinoma. Eur Arch Otorhinolaryngol. 2017;274(6):2573‐2580. 10.1007/s00405-017-4538-3 28324180

[10] Díaz‐Molina JP , Rodrigo JP , Álvarez‐Marcos C , Llorente JL , Costales M , Suárez C . Oncological results after surgical treatment of squamous cell cancer of the lateral wall of the oropharynx. Laryngoscope. 2011;121(7):1449‐1454. 10.1002/lary.21787 21647899

[11] Laccourreye O , Benito J , Garcia D , Menard M , Bonfils P , Holsinger C . Lateral pharyngotomy for selected invasive squamous cell carcinoma of the lateral oropharynx. Part II: when and why. Laryngoscope. 2013;123(11):2718‐2722. 10.1002/lary.24246 23775844

[12] Laccourreye O , Seccia V , Ménard M , Garcia D , Vacher C , Holsinger FC . Extended lateral pharyngotomy for selected squamous cell carcinomas of the lateral tongue base. Ann Otol Rhinol Laryngol. 2009;118(6):428‐434. 10.1177/000348940911800605 19663374

[13] Pang L , Jeannon JP , Simo R . Minimizing complications in salvage head and neck oncological surgery following radiotherapy and chemo‐radiotherapy. Curr Opin Otolaryngol Head Neck Surg. 2011;19(2):125‐131. 10.1097/MOO.0b013e3283440ee3 21297476

[14] Agrawal A , Wenig BL . Resection of cancer of the tongue base and tonsil via the transhyoid approach. Laryngoscope. 2000;110(11):1802‐1806. 10.1097/00005537-200011000-00005 11081588

[15] Haque R , Contreras R , McNicoll MP , Eckberg EC , Petitti DB . Surgical margins and survival after head and neck cancer surgery. BMC Ear Nose Throat Disord. 2006;6:2. 10.1186/1472-6815-6-2 16503981 PMC1395327

[16] Forastiere A , Koch W , Trotti A , Sidransky D . Head and neck cancer. N Engl J Med. 2001;345(26):1890‐1900. 10.1056/NEJMra001375 11756581

[17] Pignon JP , Maître A , Maillard E , Bourhis J . Meta‐analysis of chemotherapy in head and neck cancer (MACH‐NC): an update on 93 randomised trials and 17,346 patients. Radiother Oncol. 2009;92(1):4‐14. 10.1016/j.radonc.2009.04.014 19446902

[18] Gañán L , López M , García J , Esteller E , Quer M , León X . Management of recurrent head and neck cancer: variables related to salvage surgery. Eur Arch Otorhinolaryngol. 2016;273(12):4417‐4424. 10.1007/s00405-016-4093-3 27188507

[19] Goodwin WJ, Jr. Salvage surgery for patients with recurrent squamous cell carcinoma of the upper aerodigestive tract: when do the ends justify the means? Laryngoscope. 2000;110(3 pt 2 suppl 93):1‐18. 10.1097/00005537-200003001-00001 10714711

[20] Bachar GY , Goh C , Goldstein DP , O'Sullivan B , Irish JC . Long‐term outcome analysis after surgical salvage for recurrent tonsil carcinoma following radical radiotherapy. Eur Arch Otorhinolaryngol. 2010;267(2):295‐301. 10.1007/s00405-009-1070-0 19756684

[21] Röösli C , Studer G , Stoeckli SJ . Salvage treatment for recurrent oropharyngeal squamous cell carcinoma. Head Neck. 2010;32(8):989‐996. 10.1002/hed.21273 19953618

[22] Laccourreye O , Benito J , Menard M , Garcia D , Malinvaud D , Holsinger C . Lateral pharyngotomy for selected invasive squamous cell carcinoma of the lateral oropharynx—part I: how. Laryngoscope. 2013;123(11):2712‐2717. 10.1002/lary.24161 24325020

[23] Nichols AC , Kneuertz PJ , Deschler DG , et al. Surgical salvage of the oropharynx after failure of organ‐sparing therapy. Head Neck. 2011;33(4):516‐524. 10.1002/hed.21480 20652974

[24] Zenga J , Graboyes E , Janz T , et al. Salvage of recurrence after surgery and adjuvant therapy: a multi‐institutional study. Otolaryngol Head Neck Surg. 2019;161(1):74‐81. 10.1177/0194599819830664 30753110

[25] Agra IMG , Carvalho AL , Pontes E , et al. Postoperative complications after en bloc salvage surgery for head and neck cancer. Arch Otolaryngol Head Neck Surg. 2003;129(12):1317‐1321. 10.1001/archotol.129.12.1317 14676158

[26] Weert S , Leemans CR . Salvage surgery in head and neck cancer. Oral Dis. 2021;27(1):117‐124. 10.1111/odi.13582 32738064 PMC7821237

[27] Kostrzewa JP , Lancaster WP , Iseli TA , Desmond RA , Carroll WR , Rosenthal EL . Outcomes of salvage surgery with free flap reconstruction for recurrent oral and oropharyngeal cancer. Laryngoscope. 2010;120(2):267‐272. 10.1002/lary.20743 20013840 PMC3389788

[28] Patel SN , Cohen MA , Givi B , et al. Salvage surgery for locally recurrent oropharyngeal cancer. Head Neck. 2016;38(suppl 1):E658‐E664. 10.1002/hed.24065 25867012

